# Gendered Pathways of Internalizing Problems from Early Childhood to Adolescence and Associated Adolescent Outcomes

**DOI:** 10.1007/s10802-020-00623-w

**Published:** 2020-02-10

**Authors:** Leslie Morrison Gutman, Natasha Codiroli McMaster

**Affiliations:** 1 University College London, 1-19 Torrington Place, Londo6n, WC1E 7HB UK; 2 UCL Institute of Education, 20 Bedford Way, London, WC1H 0AL UK

**Keywords:** Internalizing problems, Developmental trajectories, Early childhood, Adolescence, Group-based modeling, Adolescent outcomes

## Abstract

Despite trends indicating worsening internalizing problems, characterized by anxiety and depression, there is dearth of research examining gender differences in developmental trajectories of internalizing problems from early childhood to adolescence. Drawing on the UK Millennium Cohort Study (*n* = 17,206, 49% female), this study examines trajectories of parent-reported, clinically-meaningful (reflecting the top 10%) internalizing problems from ages 3 to 14 years and their early predictors and adolescent outcomes. Group-based modelling revealed three trajectories when examining boys and girls together, but there were significant gender differences. When examining boys and girls separately, four trajectories were identified including two relatively stable trajectories showing either high or low probabilities of internalizing problems. An increasing trajectory was also found for both boys and girls, showing an increasing probability of internalizing problems which continued to rise for girls, but levelled off for boys from age 11. A decreasing trajectory was revealed for boys, while a moderate but stable trajectory was identified for girls. Boys and girls in the increasing and high probability groups were more likely to report a number of problematic outcomes including high BMI, self-harm, low mental wellbeing, depressive symptoms, and low educational motivation than the low group. Girls on the increasing trajectory also reported more cigarette and cannabis use and early sexual activity at age 14 compared to girls on the low trajectory. Findings suggest that intervention strategies take a systemic view, targeting not only internal feelings, but also behaviours potentially associated with later negative outcomes.

Internalizing problems, characterized by anxiety and depression, represent one of the most common forms of child psychopathology, with a higher prevalence in girls than boys (Green et al. 2005). Studies of school-age children using parent-reports as well as diagnostic tools have shown a strong, positive association between depression and generalized anxiety, suggesting that these can be classified according to a single internalizing disorder (Achenbach 1991; Moffitt et al. 2007; Sterba et al. 2007). Recent data show a population-level increase in internalizing problems, including anxiety disorders (characterised by fear and worry) and depressive disorders (characterised by sadness, loss of interest and energy, and low self-esteem), for children aged 5 to 15 years living in England, rising from 3.8% in 2004 to 5.8% in 2017 (Mental Health of Children and Young People in England, 2018). Internalizing problems in childhood and adolescence are strongly predictive of later difficulties including co-morbid mental health problems, disrupted social relationships, substance abuse, and reduced educational performance (Dekker et al. 2007; McLeod et al. 2016; Measelle et al. 2006). Despite evidence demonstrating the effectiveness of early intervention (Burt et al. 2016; Toth et al. 2016), internalizing problems in children and adolescents often are left undiagnosed and untreated (Public Health England 2016). Given the possible negative consequences as well as the potential for early intervention, it is critical to understand the development of internalizing problems from an early age. This study identifies subgroups with distinct longitudinal profiles of parent-reported internalizing problems from ages 3 to 14 years and investigates how early predictors and adolescent outcomes differentiate these trajectory groups, assessing gender differences. The identification of diverse developmental trajectories from early childhood to adolescence has important clinical implications for prevention and treatment approaches by providing insight into the pathways leading to different subtypes of internalizing difficulties.

## Trajectories of Internalizing Problems

A developmental psychopathology framework emphasizes elucidating variation in the age of onset and developmental course of normative and psychopathological development, revealing continuities and discontinuities among diverse pathways (Cicchetti and Rogosch 2002). This framework views development as an active dynamic process that can diverge depending on children’s individual characteristics and environmental contexts, showing unique patterns of change for different subgroups. Group-based trajectory modelling has enabled a more heterogeneous specification of developmental pathways than a variable-oriented approach, allowing the examination of questions relevant to developmental psychopathological theory (Cicchetti and Rogosch 2002). This person-centred approach has advanced research into externalizing and antisocial behaviour, highlighting that individuals follow distinct pathways from early childhood and through adolescence (e.g., Gutman et al. 2019; Hyde et al. 2015). Most of the research examining the development of internalizing problems has investigated the average longitudinal course, neglecting heterogeneity. Yet, an examination of subgroups with varying levels of severity and rates of change may illuminate different etiological and predictive relationships (Bauer & Curran, 2003).

A handful of studies have identified developmental trajectories of internalizing problems from childhood to adolescence (Fanti and Henrich 2010; Korhonen et al., 2014; Letcher et al. 2009; Nivard et al., 2017; Sterba et al. 2007), showing between three and six trajectory groups. Using diverse measures including the Child Behavior Checklist (CBCL) and DAWBA, all of these studies identified both high and low trajectories, showing stable levels of either high or low internalizing problems, respectively, from childhood to adolescence. Of these, the studies examining data from childhood through middle or late adolescence revealed both increasing and decreasing groups, where children show either an increase or decrease, respectively, from childhood to adolescence (Korhonen et al., 2014; Letcher et al. 2009; Nivard et al., 2017). One of these studies also examined externalizing scores and modeled their co-occurrence across childhood and adolescence, showing an association particularly when problems started early (Nivard et al., 2017). Overall, these studies suggest that there is heterogeneity in the pathways of internalizing problems from childhood to adolescence.

There are documented gender differences in the prevalence rates, developmental course, precursors, and consequences of internalizing problems (Zahn-Waxler et al. 2008). Consistent gender differences in internalizing trajectories are thought to emerge in adolescence, with girls reporting higher mean levels and a sharper increase in problems compared to boys (Leve et al. 2005). A number of hypotheses have been put forth to explain these gender differences including dispositional characteristics such as girls’ heightened reactivity and rumination styles and socialization experiences such as parents’ expectations for daughters to be more prosocial and submissive than sons (Zahn-Waxler et al. 2000). These risk factors have the potential to lead to greater internalizing problems in the face of challenges in early adolescence (Nolen-Hoeksema and Girgus 1994). Developmental models that test for possible gender differences will help elucidate whether there are distinct pathways for boys and girls and, if so, whether there are differences in their level of severity and/or age-related rates of change. Only one study examined gender-specific trajectories of internalizing problems from ages 2 to 11 years for both males and females (Sterba et al. 2007). This study found three trajectories: high, low, and decreasing/increasing, which decreased until around age 6 and then steadily increased. Although the number, prevalence, and predictive validity of the trajectories were similar for boys and girls, there were statistically significant gender differences in the initial values and rates of change. Girls were classified in the high group twice as often as were boys; while boys were twice as likely to be in the decreasing/increasing group, highlighting potential gender differentiation in trajectories of internalizing psychopathology.

There are several limitations of the available literature base. All of these studies relied on samples which were gathered before the millennium, and only one study examined gender differences. The generation born after the millennium has faced unique challenges, including the emergence of social media as a prominent pastime for adolescents. The increased accessibility and time spent on social media has raised new concerns about adolescents’ mental health (Twenge et al. 2018). Furthermore, there is a documented population-level increase in internalizing distress, particularly for girls (Collishaw et al. 2010; Fink et al. 2015; Gutman et al. 2018a), highlighting the importance of assessing gender differences in internalizing trajectories for more recently born nationally-representative samples, allowing conclusions drawn at the population-level. In addition, none of these studies have examined gender differences in internalizing problems from toddlerhood to mid-adolescence. An examination of internalizing problems from early childhood would show the emergence of gender differences in internalized pathology (Sterba et al. 2007), while following their course into adolescence would enlighten our understanding of their divergence across development. Drawing on the Millennium Cohort Study (MCS), a nationally representative sample of children born in the UK in 2000–2001, this study fills these research gaps through the identification of distinct trajectories using parent-reported internalizing problems from ages 3 to 14 years.

## Early Predictors and Adolescent Outcomes

A secondary aim is the examination of early predictors and adolescent outcomes of internalizing problems trajectories. According to the developmental psychopathology approach, diverse developmental trajectories are distinguished by different risk etiologies and associated outcomes. This provides external validation by assessing whether membership in a particular trajectory can be predicted by and predict measures other than those used to create the trajectory groups (von Eye and Bergman 2003). 

In terms of early factors, this study examines those factors that have been shown to predict heterogeneity in the development of internalizing problems from toddlerhood through adolescence including parental psychopathology, socio-economic disadvantage, low birthweight, and smoking in pregnancy (Fanti and Henrich 2010; Nivard et al., 2017; Shore et al. 2018). We extend previous findings by examining both paternal and maternal psychopathology.

Given that there is little evidence concerning how trajectories of internalizing problems from childhood to adolescence may be related to adolescent outcomes, this study explores this association among a number of relevant adolescent outcomes including problematic behaviours, mental and physical health, and relationships. For problematic behaviours, including alcohol, cigarette, substance abuse, and early sexual activity, there is evidence showing an association between these behaviours and depressive symptoms (e.g., Chaiton et al. 2009; Costello et al. 2008; Danzo et al. 2017; Skogen et al. 2016), but less is known about gender differences. In line with the “gender paradox of co-morbidities” (Loeber and Keenan 1994), girls may be less likely to engage in delinquent behaviour than boys, but when they do, they may be more likely to be depressed and anxious (Zahn-Waxler et al., 2008). Examining their associations with trajectories of internalizing problems from early childhood to adolescence will provide a better understanding of how heterogeneous groups experience different manifestations of later problematic behaviours.

There has also been increasing attention on the negative outcomes associated with social media in adolescence (Best et al. 2014; Strasburger et al. 2013; Woods and Scott 2016), with some indication that the mental health of girls may be vulnerable to its use (Booker et al. 2018). Although a moderate significant association have been found between social media and depressive symptoms in young people, most of these studies are cross-sectional or of a limited duration (Barry et al. 2017; McCrae et al. 2017). There is recent evidence that increasing use of social media is associated with increasing depressive symptoms in girls (Raudsepp and Kais 2019). However, there is little or no research examining the role of social media use in gendered pathways of internalizing problems.

Internalizing problems may also be related to the timing of puberty (Patton et al. 2008), with some evidence showing a stronger association for females than males (Lewis et al. 2015; Patton et al. 2008; Negriff and Susman 2011; Ullsperger and Nikolas 2017). Higher BMI has also been shown to be a risk factor of internalizing problems, particularly for adolescent girls (Dockray et al. 2009; Richardson et al. 2006). However, there is little research examining the role of puberty and BMI in association with heterogeneous trajectories of internalizing problems for boys and girls from early childhood to adolescence.

As a means of external validation, measures of adolescent-reported mental wellbeing and depressive symptoms are included as outcomes. Further associations among parent-reported trajectories of internalizing problems and parent-reported mental health difficulties, including conduct problems, peer problems, and hyperactivity, are examined. Lastly, based on research suggesting that positive school adjustment and better parent-child relations are associated with recovery from elevated internalizing trajectories, adolescent-reported measures of educational motivation and parent-child relationships are included as outcomes (Letcher et al. 2009).

## Current Study

Drawing on the Millennium Cohort Study (MCS), a nationally representative sample of children born in the UK in 2000–2002, this study addresses research gaps through the identification of distinct trajectories of parent-reported internalizing problems from ages 3 to 14 years and the examination of early predictors and adolescent outcomes. Unlike studies that examine gender differences in internalizing trajectories where males and females are grouped together, this study tests whether the intercepts and slopes of heterogeneous pathways differ according to gender. If statistically significant differences emerge, then distinct gendered trajectories will be identified. This is important as some pathways may be identified for one gender, but not for the other. Despite the advantages of estimating gender-specific trajectories, trajectories may be identified that are not clinically meaningful (Gutman et al., 2018b), e.g., a high trajectory group of boys that has relatively lower levels of internalizing problems than girls. To remedy this, age-based bandings using national norms from England (reflecting the top 10%), shown to strongly predict later internalizing diagnoses, are used (Meltzer et al. 2000). Measures of mental health problems tend to be highly skewed, with most individuals in the lowest category. Therefore, a clinically relevant measurement of internalizing problems may be better able to detect diverse but meaningful developmental patterns, providing an understanding of the pathways leading to clinically diagnosable internalizing disorder.

n terms of the number of trajectories, the existing literature described above suggests that four distinct trajectories may be identified for males and females. Both low and high trajectories are expected, with a higher prevalence of females in the high group (Sterba et al. 2007). Developmental change in boys and girls is also expected for better and for worse. In line with other studies (Korhonen et al., 2014; Letcher et al. 2009; Nivard et al., 2017), a decreasing group, who initially present as having a high or moderate probability of internalizing problems but shows a decrease over time, as well as an increasing group, who show a rising probability of poor internalizing health during the transition to adolescence and beyond, may be identified. However, girls may show an increasing probability of internalizing problems earlier (around age 10), as compared to boys (Kelly et al. 2016). Further, in line with prevalence rates in adolescence (Public Health England 2016), there may be a higher prevalence of females than males in the increasing group.

As shown in previous studies (Fanti and Henrich 2010; Nivard et al., 2017; Sterba et al. 2007), it is expected that early risk factors including maternal and paternal psychopathology, maternal smoking in pregnancy, and socio-economic disadvantage are associated with the high or decreasing trajectories, in comparison to the low group. Maternal psychopathology may be more strongly related to the higher problem groups for girls than boys (Zahn-Waxler et al. 2000). Given the exploratory nature of the adolescent-reported outcomes, there are no firm expectations regarding their associations, although problematic behaviours may show a stronger association with the high and increasing trajectories compared to a low group, particularly for girls in light of the “gender paradox of co-morbidity” (Loeber and Keenan 1994). Girls on the high or increasing trajectories may also be more likely to have a high BMI and use social media compared to boys on these pathways (Booker et al. 2018; Dockray et al. 2009; Richardson et al. 2006). As a means of external validation, those on the high or increasing pathways may also be more likely to report lower mental wellbeing and more depressive symptoms compared those on other trajectories. Lastly, in light of the co-morbidity among parent-reported mental health problems for children and adolescents, it is expected that those on the problematic pathways will show higher levels of parent-reported conduct problems, peer problems, and hyperactivity compared to those on the low pathway.

## Method

### Study Sample

MCS is a nationwide longitudinal study following children born in all four countries of the UK between September 2000 and January 2002 (Joshi and Fitzsimons 2016). The survey was sampled in a complex clustered and disproportionately stratified design. The clusters were electoral wards, and the strata oversampled areas of high child poverty, minority ethnic populations in England and the three smaller countries of the UK. Data are so far available from six sweeps of interviews with the families. The first survey, MCS1 (child age 9 months) was in the field mainly in 2001, fieldwork for MCS2 (age 3 years) was mainly during 2004, for MCS3 (age 5 years) mainly during 2006, and for MCS4 (age 7 years) mainly during 2008. MCS5 (age 11 years) collected data mainly in 2012 when the cohort children were in their last year of primary school. MCS6 (age 14 years) collected data mainly in 2015 when they were in secondary school. Informants were overwhelmingly mothers (more than 95%). The number of families who have been interviewed at least once is 19,243, including 692 families in England who were not recruited until MCS2. If these cases are counted, the initial response rate was 71%. In this study, the sample included one child per family, excluding children who were the second or third in sets of twins and triplets. Group-based trajectories were based on 17,880 children (girls = 8765; males = 9115) with parent ratings of internalizing problems in at least two surveys.

## Measures

### Internalizing Problems

Internalizing Problems were assessed with the emotional problems subscale of the Strengths and Difficulties Questionnaire (SDQ) (Goodman 1997, 2001), completed by the parent. The SDQ is a screening questionnaire with extensive psychometric support (www.sdqinfo.com). In the MCS, construct, convergent, discriminant, and predictive validity have been established for the SDQ subscales, showing good internal reliability, ranging from 0.75 to 0.79 at ages 3, 5, and 7 for emotional problems (Croft et al. 2015). At ages 11 and 14, alphas were 0.71 and 0.73, respectively. The questionnaire assesses emotional problems in the past 6 months using five items including “many fears, easily scared”, “often unhappy, down-hearted or tearful”, and “many worries, often seems worried” (0 = not true, 1 = somewhat true, 2 = certainly true). These scores are totalled with a range of 0 to 10, with parents reporting a mean score of 2.04 (SD = 2.14) at age 3, 1.40 (SD = 1.61) at age 5, 1.54 (SD = 1.77) at age 7, 1.87 (SD = 2.00) at age 11, and 2.05 (SD = 2.14) at age 14. To ensure that these levels are clinically meaningful, SDQ bandings were used based on externally given UK norms at each age (Meltzer et al. 2000), where 10% in that reference sample with the highest scores were considered to be at high risk of emotional problems (0 = not high risk; 1 = high risk). Using those SDQ bandings in this sample, 9.22% (SD = 0.30) of the children were considered to be high risk of conduct problems with a mean score for the totalled emotional problems subscale of 4.91 (SD = 1.25) at age 3, 5.61% (SD = 0.23) with a mean score of 5.81 (SD = 1.16) at age 5, 7.64% (SD = 0.27) at age 7 with a mean score of 5.95 (SD = 1.22), 11.13% (SD = 0.31) with a mean score of 6.14 (SD = 1.31) at age 11, and 13.76% (SD = 0.34) with a mean score of 6.24 (SD = 1.42) at age 14.

### Early Predictors

All early predictors were measured when the child was 9 months-old. These include: race/ethnicity (0 = White British; 1 = Black and Minority Ethnicity (BME), teenage mother (1 = mother 19 years or younger at the child’s birth; 0 = older than 19 years), low birthweight (1 = less than 2.5 kg; 0 = other), single parent families (1 = single parent; 0 = two-parent family), parental education (1 = no qualifications or qualifications below General Certificate of Secondary Education (GCSE) level; 0 = qualifications at or above GCSE level), parental income (1 = lowest income quintile; 5 = highest quintile), whether they lived in social housing (1 = yes; 0 = no), and whether their mother smoked during pregnancy (1 = yes, 0 = no).

Maternal and paternal depressive symptoms (alpha = 0.72 for mothers; 0.66 for fathers) were also measured using a 9-item count variable as reported in Johnson et al. (2015) derived from the (24 item) Malaise Inventory (Rutter et al. 1970). Mothers and fathers answered such questions as “everything gets on my nerves” and “I often feel miserable or depressed” (1 = yes, 0 = no). Mothers and fathers with a score of 5 or more were considered at risk of depression (Rodgers et al. 1999).

### Adolescent-Reported Outcomes

A number of single items at age 14 years were analysed, taken from the young persons’ self-completed questionnaire. Items include: early menarche for females (0 = age 11 or older; 1 = before age 11); how many times they had an alcoholic drink in the last 12 months (0 = never, 1 = 1–2 times, 2 = 3–5 times, 3 = 6–9 times, 4 = 10–19 times, 5 = 20–39 times or 6 = 40 times or more); how often they smoked cigarettes (0 = never, 1 = only tried smoking, 2 = used to smoke, 3 = sometimes smoke, 4 = usually 1–6 cigarettes a week, 5 = usually more than 6 cigarettes a week); how often they smoked cannabis (0 = never, 1 = 1–2 times, 2 = 3–4 times, 3 = 5–10 times, 4 = 10+ times); whether they had a high BMI (0 = other; 1 = 85th percentile or higher); how many hours they spent on social networks per week (0 = none, 1 = less than half an hour, 2 = half an hour to 1 hour, 3 = 1 to 2 hours, 4 = 2 to 3 hours, 5 = 3 to 5 hours, 7 = 6 to 7 hours, 7 = 7 hours or more), and whether they ever self-harmed (0 = no; 1 = yes). They were also asked if they had engaged in any sexual activity in the past 12 months (0 = no; 1 = yes). Finally, young people were asked how often they argued with their mother and father (1 = hardly ever, never; 2 = less than once a week; 3 = more than once a week; 4 = most days).

Three additional measures were taken from the young persons’ self-completed questionnaire. Mental wellbeing was assessed using a measure developed for the youth survey of the British Household Panel Study in the 1990s (Taylor et al. 2010). This consists of a six-item scale including questions about their satisfaction with different areas of their life, including schoolwork, appearance, family, friends, school, and life as a whole. Responses were on a 1 (completely happy) to 7 (not at all happy) scale. The mean of responses was calculated for children’s overall wellbeing score, and responses were reverse coded so that a higher score represented higher wellbeing (alpha = 0.86).

For depressive symptoms, the shortened-version of the Moods and Feelings Questionnaire (MFQ) was used. As a screening tool for depression, this measure consists of 13 descriptive phrases about how they had been acting and feeling recently (Angold et al. 1995), such as: “I felt miserable or unhappy”, “I didn’t enjoy anything at all”, and “I felt so tired I just sat around and did nothing” (1 = not true, 2 = sometimes, 3 = true), and the mean of responses was used , with higher scores representing more negative feelings (alpha = 0.93).

To assess low educational motivation, the following question responses were combined: “How often do you try your best at school?”, “How often do you find school interesting?” (reverse-coded), “How often do you feel unhappy at school?”, “How often do you get tired at school?”, “How often do you feel school is a waste of time?”, and “How often difficult to keep mind on work at school?” Responses ranged from all of the time (1) to never (4), and the mean of responses was calculated (alpha = 0.75).

### Parent-Reported Outcomes

Parent-reported conduct problems, peer problems, and hyperactivity at age 14 were assessed by the SDQ (Goodman 1997, 2001). Alphas are 0.64, 0.63, and 0.78 respectively. The questionnaire assesses mental health problems in the past 6 months using five items for each subscale. Example questions include “often lies or cheats” for conduct problems, “rather solitary, tends to play alone” for peer problems and “easily distracted, concentration wanders” for hyperactivity. SDQ bandings based on externally given UK norms at each age were used (Meltzer et al. 2000), where 10% in that reference sample with the highest scores were considered to be at high risk of mental health problems (0 = not high risk; 1 = high risk).

### Statistical Analyses

Group-based trajectory analysis in STATA TRAJ (Jones and Nagin 2013) was used to identify discrete groups of children following similar progressions of internalizing problems as a function of age measured in months at each interview. Group-based trajectory modelling is a specialized form of finite mixture modelling (see Nagin 2005; Nagin and Odgers 2010). Full Information Maximum Likelihood (FIML) estimated the model parameters, thereby including every case with at least two parental ratings (Schafer and Graham 2002). Binary logit distribution was specified as internalizing problems are considered a dichotomous variable (e.g., whether clinically meaningful or not). To establish the best fitting solution, a range of fit indicators was examined, including the lowest absolute Bayesian Information Criterion (BIC) (Nagin 2005), the average posterior probability of group membership (0.70 being acceptable), and a close correspondence between the estimated probability of group membership and the proportion assigned to that group based on the posterior probability of group membership. To assess whether gender differences were evident in the intercept and slope of the trajectories, gender and time-varying gender by age covariates were included in the model (Jones and Nagin 2013).

In order to account for the complex clustered and stratified survey design of MCS, *svy* in STATA was used in the following stages of the analyses. First, gender differences in internalizing problems, early predictors, and adolescent outcomes were assessed using univariate regressions for each predictor on gender. For significant differences, the effect size using Cohen’s *d* is reported. Then, the proportions and standard deviations of the early predictors and adolescent outcomes by the assigned trajectory group were examined (see Tables 2 and 3). To do this, univariate regressions were run for each factor on trajectory group status and then post-hoc tests were conducted to compare all possible pairwise differences among the four groups using the Bonferroni correction.

Sampling weights reflecting the MCS design were used in the group-based trajectory modelling and subsequent analyses to correct for disproportionate sampling. The sampling weights reduce the apparent size of cells populated by oversampled strata, such as minority ethnic populations and increase the apparent size of strata with under-sampled cases. For the subsequent analyses, attrition weights were applied to restore the social profile of the whole cohort. The MCS survey team has developed attrition weights to correct for biases due to non-response (Hansen 2014).

## Results

### Gender Differences in Internalizing Problems, Predictors and Outcomes

Results for girls and boys are presented separately, and effect sizes for statistically significant differences are shown (see Table 1). Although incidence of parent-reported internalizing problems was similar amongst girls and boys in most age groups, at age 14, girls were significantly more likely to have internalizing problems compared to boys. Girls were also more likely to have a low birthweight. In terms of adolescent-reported outcomes, girls were more likely to report smoking tobacco, self-harming, and spending time on social media. Girls also reported lower mental wellbeing, more depressive symptoms, and more arguments with their mothers than boys, while boys reported lower educational motivation compared to girls. Parent-reported conduct problems, peer problems, and hyperactivity were all higher for boys than girls.

**Table 1:** Gender differences among internalizing problems, early predictors and adolescent outcomes

Measures	Boys		Girls		F-test	Cohen’s d
	Mean(SD)	95% CI	Mean(SD)	95% CI		
Internalizing problems						
Probability at age 3	0.09(0.28)	(0.08–0.10)	0.09(0.28)	(0.08–0.09)	F(1, 389) = 0.12	
Probability at age 5	0.05(0.23)	(0.05–0.06)	0.06(0.23)	(0.05–0.06)	F(1, 389) = 0.05	
Probability at age 7	0.08(0.28)	(0.08–0.09)	0.08(0.27)	(0.07–0.08)	F(1, 389) = 1.55	
Probability at age 11	0.12(0.32)	(0.11–0.13)	0.12(0.33)	(0.11–0.13)	F(1, 389) = 1.44	
Probability at age 14	0.12(0.33)	(0.11–0.13)	0.18(0.38)	(0.16–0.19)	F(1, 389) = 67.85***	−0.15
Early predictors						
BME background	0.11(0.31)	(0.09–0.13)	0.12(0.32)	(0.09–0.14)	F(1, 389) = 1.06	
Teenage mother	0.03(0.17)	(0.02–0.03)	0.03(0.17)	(0.02–0.03)	F(1, 389) = 0.17	
Low birth weight	0.06(0.24)	(0.06–0.07)	0.07(0.26)	(0.07–0.08)	F(1, 389) = 11.05***	−0.05
Single parent	0.15(0.35)	(0.13–0.16)	0.14(0.35)	(0.13–0.15)	F(1, 389) = 0.85	
Parent low qualifications	0.19(0.39)	(0.18–0.20)	0.18(0.38)	(0.17–0.19)	F(1, 389) = 0.35	
Income quintile	3.00(1.41)	(2.92–3.08)	3.00(1.42)	(2.92–3.07)	F(1, 389) = 0.13	
Social housing	0.23(0.42)	(0.21–0.25)	0.24(0.43)	(0.22–0.26)	F(1, 389) = 0.17	
Maternal smoking in pregnancy	0.22(0.42)	(0.21–0.24)	0.21(0.41)	(0.19–0.22)	F(1, 389) = 3.69	
Maternal depressive symptoms	0.08(0.27)	(0.07–0.08)	0.07(0.26)	(0.07–0.08)	F(1, 389) = 3.31	
Paternal depressive symptoms	0.05(0.21)	(0.04–0.05)	0.05(0.22)	(0.04–0.06)	F(1, 389) =1.41	
Self-reported adolescent outcomes						
Early menarche	n/a	n/a	0.24(0.43)	(0.22–0.25)	n/a	
How many times had an alcoholic drink	0.90(1.31)	(0.85–0.96)	0.85(1.24)	(0.80–0.90)	F(1, 389) = 4.98	
How often do you smoke	0.30(0.87)	(0.27–0.33)	0.39(1.01)	(0.35–0.42)	F(1, 389) = 16.92***	−0.08
How often smoke cannabis	0.12(0.57)	(0.10–0.14)	0.10(0.50)	(0.09–0.12)	F(1, 389) = 2.79	
Ever self-harmed	0.09(0.29)	(0.08–0.10)	0.22(0.41)	(0.21–0.24)	F(1, 389) = 356.80***	−0.35
High BMI	0.16(0.37)	(0.15–0.17)	0.16(0.36)	(0.14–0.17)	F(1, 389) = 0.00	
Hours spent on social networks	3.01(2.15)	(2.92–3.11)	4.05(2.07)	(3.97–4.13)	F(1, 389) = 752.28***	−0.51
Engaged in sexual activity	0.03(0.18)	(0.03–0.04)	0.03(0.17)	(0.03–0.04)	F(1, 389) = 0.36	
Mental wellbeing	5.62(1.04)	(5.58–5.65)	5.26(1.16)	(5.21–5.30)	F(1, 389) = 280.09***	0.31
Depressive symptoms	1.34(0.38)	(1.32–1.35)	1.55(0.51)	(1.53–1.57)	F(1, 389) = 657.26***	−0.48
Low educational motivation	2.90(0.48)	(2.89–2.92)	2.86(0.53)	(2.83–2.88)	F(1, 389) = 20.77***	0.08
How often argue with mother	2.75(1.04)	(2.71–2.78)	2.91(1.06)	(2.87–2.94)	F(1, 389) = 86.35***	−0.17
How often argue with father	2.44(1.04)	(2.39–2.48)	2.45(1.03)	(2.41–2.49)	F(1, 389) = 4.36	
Parent-reported adolescent outcomes						
Conduct problems	0.15(0.36)	(0.14–0.17)	0.11 (0.32)	(0.10–0.12)	F(1, 389) = 29.80***	0.08
Peer problems	0.20(0.40)	(0.19–0.22)	0.16 (0.37)	(0.15–0.18)	F(1, 389) = 18.86***	0.07
Hyperactivity	0.15(0.36)	(0.14–0.17)	0.08 (0.27)	(0.07–0.09)	F(1, 389) = 93.68***	0.22

F-tests were conducted using svy in STATA, which reports design degrees of freedom for a complex clustered and stratified survey design. **p < 0.01; ***p < 0.001

### Trajectories of Internalizing Problems

**Fig. 1:**
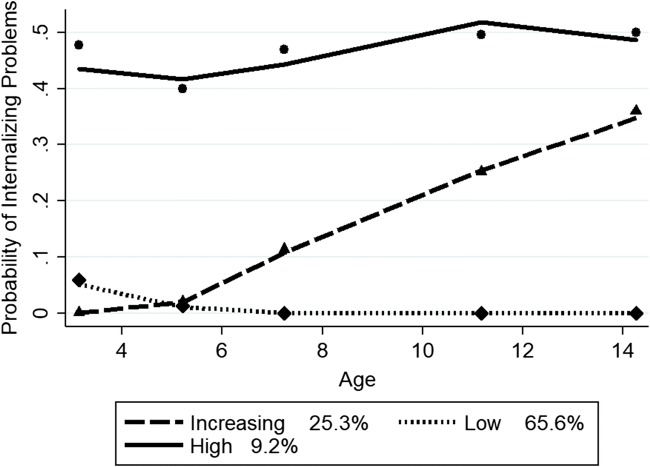
Trajectory groups of internalizing problems, both boys and girls. *Note*. Shown are estimated trajectories (lines), observed group means at each age (markers) and estimated group percentages

Group-based trajectory analysis was first run with both boys and girls together. Models with three to five trajectories with linear to quadratic functional forms were examined. The three-group, quadratic model fit the data best. The BIC score for the three group, quadratic model (−18,404.5) had the absolute lowest score compared to the four (−18,626.37) and five (−18,486.06) group, quadratic models. The mean posterior probability scores ranged from 0.78 to 0.82 for the three-trajectory model, with a mean of 0.80, indicating that most children fit their assigned trajectory well. Figure 1 depicts the probability of clinically relevant internalizing problems for the three trajectory groups from ages 3 to 14 years, along with the estimated proportion in each group. The predicted and observed means were close, indicating a good fit of the model. There were low (65.6% estimated; 66.4% actual), high (9.2% estimated; 8.5% actual), and increasing (23.5% estimated; 25.1% actual) probability groups. Gender differences in the intercept and slope of these trajectories were tested using gender, time-varying gender by age (linear slope), and time-varying gender by age-squared (quadratic) covariates. These findings revealed significant differences in the intercept, linear, and quadratic slopes, where *p* < 0.0001, for the high and increasing probability groups. Thus, group-based trajectory analysis was run for boys and girls, separately.

**Fig. 2:**
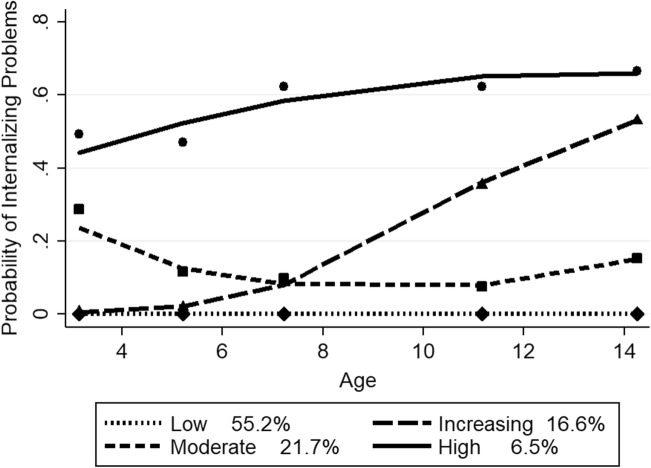
Girls’ trajectory groups of internalizing problems. *Note*. Shown are estimated trajectories (lines), observed group means at each age (markers) and estimated group percentages

For girls, the four-group, quadratic model fit the data best. The BIC score for the four group, quadratic model (−9840.27) had the absolute lowest score compared to the three (−9955.57) and five (−9852.91) group, quadratic models. The mean posterior probability scores for girls ranged from 0.72 to 0.78 for the four-group trajectory model, with a mean of 0.74, indicating that most girls fit their assigned trajectory well. Figure 2 depicts the probability of clinically relevant internalizing problems for the four trajectory groups in girls from ages 3 to 14 years, along with the estimated proportion in each group. The predicted and observed means were close, indicating a good fit of the model. The low problem group (55.2% estimated; 56.5% actual) displayed a near zero probability of internalizing problems from ages 3 to 14. The increasing group (16.6% estimated, 16.7% actual) demonstrated a near zero probability in early childhood and then showed an increase from ages 5 to 14, rising to more than 50%. A moderate group (21.7% estimated; 20.8% actual) followed a probability of above 0.20 from age 3, decreasing to 0.10 from age 5 and remaining fairly stable until age 14, when there was a slight increase to almost 0.20. In the high group, a small percentage of girls (6.5% estimated; 6.1% actual) showed a relatively high probability of close to 0.40 at age 3, increasing until age 11, reaching more than 60% .

**Fig. 3:**
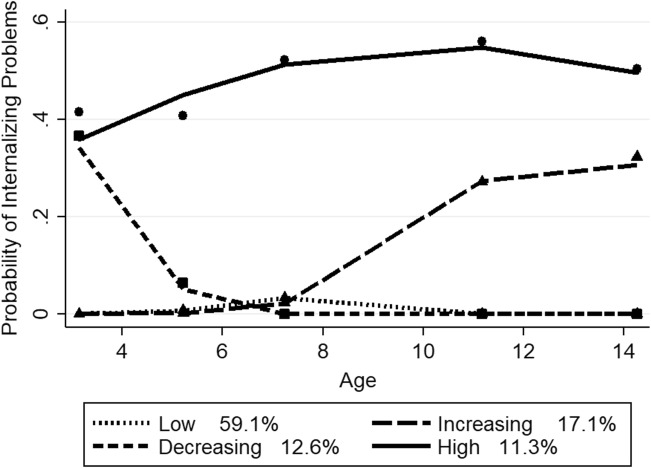
Boys’ trajectory groups of internalizing problems. *Note*. Shown are estimated trajectories (lines), observed group means at each age (markers) and estimated group percentages

For boys, the final model meeting the selection criteria also included four quadratic trajectories. The BIC score for the four-group model (−9394.62) is lower compared to the three (−9398.60) and five (−9409.03) group models. The mean posterior probability scores ranged from 0.72 to 0.88 for the four-trajectory model, with a mean of 0.80, indicating that most boys fit their assigned trajectory well. Figure 3 depicts the probability of clinically relevant internalizing problems for the four trajectory groups in boys from ages 3 to 14, along with the estimated percentage in each group. The predicted and observed values had a high level of correspondence, indicating a good fit of the model. The low problem group (59.1% estimated; 60.05% actual) showed an almost zero probability of internalizing problems from ages 3 to 14. There was a decreasing group (12.6% estimated; 12.3% actual), which displays a high probability at age 3 (close to 40%), declining sharply to near zero by age 7 and remaining low thereafter. There was also a moderately, increasing group (17.1% estimated; 17.7% actual) showing a low probability from age 3, increasing sharply from ages 7 to 11, and levelling off to around 30% from age 11. The high group (11.3% estimated; 10.5 actual) displayed a high probability from ages 3 to 14 (around 50%), showing a steady increase up to age 7, then a slight decline from ages 11 to 14.

### Early Predictors and Adolescent Outcomes

Table 2 presents the mean differences in early risk factors and adolescent- and parent-reported outcomes among trajectory groups for girls. Girls in the high probability group generally showed more early risks than girls in the low group, with the increasing and moderate groups showing intermediate levels of some early risks. There were no significant differences among the groups for having a teenage mother and paternal psychopathology. The moderate group was disproportionally from BME backgrounds compared to the low and increasing groups. For the adolescent outcomes, girls in the increasing and high groups were more likely to report self-harm, lower mental wellbeing, more depressive symptoms, lower educational motivation, and more arguments with their mother compared to girls in the low or moderate groups, and high BMI compared to girls in the low group. Parents of girls in the increasing or high groups reported that their daughters showed more conduct problems, peer problems, and hyperactivity compared to parents of girls in the low or moderate groups, while parents of girls in the moderate group reported that their daughters had more peer problems compared to those in the low group. Girls in the increasing group further reported more early sexual activity than girls following the low or moderate pathways, more cigarette and cannabis use than girls following the low pathway, and more alcoholic use than girls following the moderate pathway. There were no significant differences among the groups in early menarche, social media use, or arguments with their father.

**Table 2:** Mean differences in early predictors and adolescent outcomes by trajectory group for girls

Variables	Trajectory group								*F*-test
	Low		Increasing		Moderate		High		
	Mean	SD	Mean	SD	Mean	SD	Mean	SD	
Early predictors									
BME background	0.11^a^	0.32	0.10^a^	0.30	0.19^b^	0.39	0.14^ab^	0.35	F(3, 387) = 4.38**
Teenage mother	0.03	0.16	0.03	0.17	0.04	0.19	0.04	0.21	F(3, 387) = 1.20
Low birth weight	0.07^a^	0.25	0.11^b^	0.31	0.09^ab^	0.29	0.07^ab^	0.26	F(3, 387) = 4.55**
Single parent	0.13^a^	0.34	0.15^ab^	0.35	0.18^bc^	0.39	0.25^c^	0.43	F(3, 387) = 9.17***
Parent low qualifications	0.17^a^	0.37	0.21^b^	0.41	0.21^ab^	0.41	0.28^b^	0.45	F(3, 387) = 8.96***
Income quintile	3.10^a^	1.41	2.83^b^	1.38	2.47^c^	1.38	2.48^c^	1.33	F(3, 387) = 38.14***
Social housing	0.22^a^	0.41	0.26^ab^	0.44	0.32^b^	0.47	0.42^c^	0.49	F(3, 387) = 18.98***
Maternal smoking in pregnancy	0.19^a^	0.39	0.24^b^	0.43	0.27^b^	0.44	0.30^b^	0.46	F(3, 387) = 7.57***
Maternal depressive symptoms	0.05^a^	0.23	0.12^b^	0.33	0.14^bc^	0.35	0.20^c^	0.40	F(3, 387) = 23.96***
Paternal depressive symptoms	0.05	0.21	0.07	0.25	0.08	0.28	0.10	0.30	F(3, 387) = 3.56
Self-reported adolescent outcomes									
Early menarche (before 11 years old)	0.23	0.42	0.24	0.43	0.27	0.45	0.27	0.45	F(3, 387) = 1.18
How many times had an alcoholic drink	0.86^ab^	1.25	0.94^a^	1.27	0.68^b^	1.12	0.82^ab^	1.22	F(3, 387) = 3.41**
How often do you smoke cigarettes	0.33^a^	0.93	0.60^b^	1.26	0.41^ab^	1.01	0.46^ab^	0.98	F(3, 387) = 6.03***
How often smoke cannabis	0.08^a^	0.46	0.20^b^	0.65	0.10^ab^	0.44	0.10^ab^	0.55	F(3, 387) = 3.28**
Ever self-harmed	0.18^a^	0.39	0.37^b^	0.48	0.17^a^	0.38	0.36^b^	0.48	F(3, 387) = 24.03***
High BMI	0.14^a^	0.35	0.20^b^	0.40	0.16^ab^	0.37	0.23^b^	0.42	F(3, 387) = 4.91**
Hours spent on social networks	4.08	1.95	4.01	2.40	3.82	2.23	4.22	2.13	F(3, 387) = 1.38
Engaged in sexual activity	0.03^a^	0.16	0.06^b^	0.24	0.02^a^	0.15	0.03^ab^	0.16	F(3, 387) = 2.98**
Mental wellbeing	5.39^a^	1.09	4.76^b^	1.28	5.31^a^	1.20	4.84^b^	1.20	F(3, 387) = 53.64***
Depressive symptoms	1.49^a^	0.48	1.79^b^	0.58	1.46^a^	0.46	1.73^b^	0.54	F(3, 387) = 49.15***
Low educational motivation	2.91^a^	0.51	2.69^b^	0.55	2.87^a^	0.55	2.69^b^	0.56	F(3, 387) = 33.75***
How often argue with mother	2.86^a^	1.03	3.09^b^	1.10	2.79^a^	1.10	3.16^b^	1.17	F(3, 387) = 10.75***
How often argue with father	2.43	0.99	2.56	1.15	2.36	1.08	2.57	1.22	F(3, 387) = 2.57
Parent-reported adolescent outcomes									
Conduct problems	0.07^a^	0.25	0.23^b^	0.42	0.13^a^	0.34	0.28^b^	0.45	F(3, 387) = 37.54***
Peer problems	0.08^a^	0.28	0.38^b^	0.49	0.19^c^	0.39	0.45^b^	0.50	F(3, 387) = 92.70***
Hyperactivity	0.04^a^	0.20	0.18^b^	0.38	0.08^a^	0.27	0.25^b^	0.43	F(3, 387) = 37.17***

F-tests and post-hoc analysis were conducted using svy in STATA, which reports design degrees of freedom for a complex clustered and stratified survey design. Post-hoc analyses using Bonferroni’s method identified significant pairwise comparisons (*p* < 0.05) between groups, shown when group means do not share any similar superscripts*.* *p < 0.05, ** *p* < 0.01, ***p < 0.001

Table 3 presents the mean differences in early risk factors and adolescent- and parent-reported outcomes among trajectory groups for boys. Boys in the high group generally showed more early risks than the low group, with the increasing and decreasing groups showing moderate early risks, for the most part. There was an overrepresentation of boys from BME backgrounds in the high and decreasing groups. No significant differences were shown for having a teenage mother and paternal psychopathology. In terms of adolescent-reported outcomes, boys in the high or increasing groups were more likely to report cigarette use, self-harm, high BMI, low mental wellbeing, and low educational motivation compared to boys in the low group and depressive symptoms compared to boys in the low or decreasing groups. The increasing group reported more arguments with their mother than the low group. Parents of boys in the increasing or high groups reported that their sons showed more conduct problems, peer problems, and hyperactivity compared to those in the low or moderate groups. There were no significant differences among the groups in alcohol use, smoking cannabis, social media use, sexual activity, and arguing with their father.

**Table 3:** Mean differences in early predictors and adolescent outcomes by trajectory group for boys

Variables	Trajectory group								*F*-test
	Low		Increasing		Decreasing		High		
	Mean	SD	Mean	SD	Mean	SD	Mean	SD	
Early predictors									
BME background	0.11^a^	0.31	0.10^a^	0.31	0.17^b^	0.37	0.16^b^	0.36	F(3, 387) = 5.30**
Teenage mother	0.03	0.16	0.03	0.18	0.07	0.25	0.04	0.20	F(3, 387) = 2.72
Low birth weight	0.06^a^	0.24	0.06^a^	0.24	0.07^ab^	0.26	0.12^b^	0.32	F(3, 387) = 5.30**
Single parent	0.14^a^	0.34	0.15^a^	0.36	0.18^ab^	0.38	0.25^b^	0.43	F(3, 387) = 11.65***
Parent low qualifications	0.18^a^	0.38	0.20^ab^	0.40	0.26^b^	0.44	0.26^b^	0.44	F(3, 387) = 6.62***
Income quintile	3.07^a^	1.40	2.94^ab^	1.45	2.69^b^	1.39	2.35^c^	1.32	F(3, 387) = 41.72***
Social housing	0.21^a^	0.41	0.28^b^	0.45	0.31^bc^	0.46	0.38^c^	0.48	F(3, 387) = 20.95***
Maternal smoking in pregnancy	0.21^a^	0.41	0.26^ab^	0.44	0.27^ab^	0.44	0.31^b^	0.46	F(3, 387) = 7.11***
Maternal depressive symptoms	0.06^a^	0.24	0.11^b^	0.31	0.15^bc^	0.36	0.17^c^	0.38	F(3, 387) = 24.36***
Paternal depressive symptoms	0.05	0.21	0.05	0.22	0.07	0.26	0.06	0.24	F(3, 387) = 1.30
Self-reported adolescent outcomes									
How many times had an alcoholic drink	0.92	1.30	0.89	1.44	0.86	1.29	0.78	1.26	F(3, 387) = 1.12
How often do you smoke	0.25^a^	0.76	0.43^b^	1.03	0.45^ab^	1.12	0.55^b^	1.28	F(3, 387) = 6.99***
How often smoke cannabis	0.10	0.53	0.15	0.59	0.13	0.60	0.23	0.78	F(3, 387) = 1.93
Ever self-harmed	0.08^a^	0.27	0.16^b^	0.36	0.08^ab^	0.27	0.14^b^	0.35	F(3, 387) = 6.90***
High BMI	0.14^a^	0.35	0.25^b^	0.43	0.15^ab^	0.36	0.22^b^	0.41	F(3, 387) = 7.02***
Hours spent on social networks	3.06	1.98	2.96	2.12	2.95	1.97	2.70	3.29	F(3, 387) = 0.71
Engaged in sexual activity	0.03	0.17	0.04	0.21	0.03	0.18	0.04	0.20	F(3, 387) = 0.38
Mental wellbeing	5.68^a^	1.02	5.38^b^	1.05	5.53^ab^	1.02	5.39^b^	1.09	F(3, 387) = 14.72***
Depressive symptoms	1.31^a^	0.36	1.45^b^	0.43	1.30^a^	0.33	1.46^b^	0.44	F(3, 387) = 17.34***
Low educational motivation	2.96^a^	0.45	2.84^b^	0.51	2.91^ab^	0.43	2.83^b^	0.53	F(3, 387) = 10.28***
How often argue with mother	2.72^a^	1.02	2.98^b^	1.04	2.74^ab^	1.06	2.75^ab^	1.21	F(3, 387) = 5.73***
How often argue with father	2.42	1.01	2.60	1.09	2.36	1.02	2.41	1.31	F(3, 387) = 2.95
Parent-reported adolescent outcomes									
Conduct problems	0.10^a^	0.30	0.33^b^	0.47	0.12^a^	0.33	0.39^b^	0.49	F(3, 387) = 54.72***
Peer problems	0.13^a^	0.33	0.47^b^	0.50	0.15^a^	0.36	0.51^b^	0.50	F(3, 387) = 87.61***
Hyperactivity	0.10^a^	0.30	0.34^b^	0.47	0.12^a^	0.32	0.36^b^	0.48	F(3, 387) = 50.69***

F-tests and post-hoc analysis were conducted using svy in STATA, which reports design degrees of freedom for a complex clustered and stratified survey design. Post-hoc analyses using Bonferroni’s method identified significant pairwise comparisons (*p* < 0.05) between groups, shown when group means do not share any similar superscripts*.* *p < 0.05, ** *p* < 0.01, ***p < 0.001

## Discussion

There is a dearth of recent research examining gender differences in pathways of internalizing problems from early childhood to adolescence. An understanding of clinically meaningful pathways for boys and girls born around the millennium is important for intervention purposes, in order to target high risk children during critical points in their development. Using evidence from a current, nationally representative UK cohort study, following the lives of over 17,000 children born in 2000/2, this study identifies distinct trajectories of internalizing problems for boys and girls from ages 3 to 14 years. Although initial findings revealed three pathways of internalizing problems when both genders were examined together, significant gender differences were shown in the intercepts and slopes of the high and increasing trajectories. When examining boys and girls separately, four trajectories were identified including two relatively stable trajectories showing either high or low probabilities of internalizing problems. An increasing trajectory was also found for both boys and girls, showing an increasing probability of internalizing problems which continued to rise for girls, but levelled off for boys from age 11. A decreasing trajectory was revealed for boys, while a moderate but stable trajectory was identified for girls. Significant early risk factors and adolescent outcomes differed among the trajectory groups. Boys and girls in the increasing and high probability groups were more likely to report high BMI, self-harm, low mental wellbeing, depressive symptoms, and low educational motivation than the low group. Girls, but not boys, on the increasing trajectory also reported more cigarette and cannabis use and early sexual activity at age 14 than girls following the low pathway. These findings suggest that the course of internalizing problems varies for boys and girls with distinct manifestations of risk.

### Trajectories of Internalizing Problems

As other trajectory-group studies of internalizing problems have shown (Fanti and Henrich 2010; Korhonen et al., 2014; Letcher et al. 2009; Nivard et al., 2017; Sterba et al. 2007), findings revealed both high and low problem groups. As expected, the low-problem group had a slightly higher prevalence of boys than girls (59% compared to 55%). In line with Sterba et al. (2007), a high problem group was revealed for both genders, showing an early-onset in childhood. Although Sterba et al. (2007) found higher prevalence rates for females than males in the high group, this study found the opposite. Unexpectedly, the prevalence rate was higher for boys than girls (11.3% versus 6.5%) in the high group. This difference may be due to the longer age range of the current study, in comparison to the earlier study, which examined trajectories up to age 11 (Sterba et al. 2007). As these data extend from early childhood to adolescence, they may be better able to capture the nuances of these diverse pathways, as well as identify when gender differences emerge in development. What these data demonstrate are a group of males and females, with a high and persistent probability of internalizing problems from an early age. Males are especially at high risk of being in this group, which may represent the preponderance of males in this cohort with special educational needs and co-morbid mental health problems, more generally (Gutman et al. 2015).

Unlike other studies which found a higher prevalence of girls in the increasing group (Nivard et al., 2017), this study found that boys and girls had a similar prevalence in a clinically meaningful increasing pathway (17.1% and 16.6%, respectively), but each showed a somewhat different trajectory shape. From a near zero probability, girls in this group showed an onset at age 5, increasing to almost 60% at age 14; whereas boys in this group showed a later onset at age 7, increasing to 30% by age 14. Thus, adolescent girls showed almost twice the likelihood of having severe internalizing problems compared to boys in this group. Similarly, Sterba et al. (2007) found that the increasing group of girls reached a higher level of internalizing problems, in comparison to the same group of males at age 11. For both genders, the increase shown at age 11 likely coincides with the onset of puberty. For girls, the probability of severe internalizing problems continued to rise, reaching levels close to the high group by age 14. For boys, the probability seemed to level off around age 11. This suggests that boys in this group show increasing but moderate vulnerability to internalizing problems, coinciding with the transition into secondary school. Girls, on the other hand, may become more susceptible to internalizing problems in mid-adolescence in line with recent data (Mental Health of Children and Young People in England, 2018), culminating in high-risk group of adolescent girls.

The findings revealed a decreasing group for males, showing a high probability of internalizing problems, close to 40% at age 3, which plunged to near zero levels thereafter. This suggests that there is a group of males who show severe internalizing problems early in childhood, maturing out of these internalizing difficulties once they reach school age. As discussed below, this group showed no evidence of higher externalizing behaviours in adolescence compared to the low group. Girls presented a moderate group, where they began with a moderate probability, showing a mild dip in childhood, with a slight increase from ages 11 to 14, coinciding with the pubertal transition. These girls show moderate probability of internalizing problems throughout childhood and adolescence, hovering between 10% and 20%. This trajectory is similar to the decreasing/increasing trajectory shown in Sterba et al. (2007), which was hypothesised to be more sensitive to environmental stressors and sensitive periods than those in the elevated, stable trajectory. In contrast to previous studies suggesting that gender differences in internalizing problems begin in adolescence (Leve et al. 2005), these findings indicate that gender differences may emerge for distinct trajectories in early childhood, in addition to those surfacing in adolescence.

### Early Predictors and Adolescent Outcomes

Early predictors and later adolescent outcomes distinguished these trajectories. As other studies have shown (Fanti and Henrich 2010; Nivard et al., 2017; Sterba et al. 2007), both genders on the high pathway experienced more early risks, including parents with lower education and income, living in social housing and with a single parent, and having a mother who smoked in pregnancy and reported more post-natal depressive symptoms than those in the low group. Boys in the high group were also more likely to have a low birthweight and BME background than boys in the low group. Both boys and girls in this group had worse adolescent outcomes, including a high BMI, self-harm, low mental wellbeing, more depressive symptoms, and low educational motivation compared to those on the low pathway, highlighting the educational, mental, and physical health risks for this group. Parents also reported higher probabilities of adolescent conduct problems, peer problems, and hyperactivity than the low or decreasing pathways. There were a few gender differences. Boys were more likely to report smoking cigarettes, while girls were more likely to report arguing with their mother, but not their father, than the low group. Nevertheless, unlike studies examining pathways of depressive symptoms (Costello et al. 2008; Danzo et al. 2017; Skogen et al. 2016), the high group did not report drinking more alcohol or using more cannabis than the low group.

Similar to previous studies, the increasing group were more likely to have mothers with post-natal depressive symptoms than the low group (Nivard et al., 2017; Sterba et al. 2007). Boys on this pathway were also more likely to live in social housing, while girls on the increasing trajectory were more likely to experience social disadvantage, in terms of low parental income and educational qualifications, have mothers who smoked during pregnancy, and have a low birthweight compared to the low group. Both boys and girls on the increasing pathway reported worse adolescent outcomes than those on the low pathway, including high BMI, self-harm, low mental well-being, more depressive symptoms, and low educational motivation; while parents reported higher probabilities of conduct problems, peer problems, and hyperactivity compared to the low or decreasing/moderate pathways. Boys on this trajectory reported greater conflict with their mothers than the low group, while girls reported more cigarette and cannabis use than the low group, more early sexual activity than the low or moderate groups, and more alcoholic use than the moderate group. These findings contribute to our understanding of the possible gender differences in both the etiology and outcomes of the increasing pathway, indicating that girls on the increasing trajectory are not only distinguished by having greater early social disadvantage compared to boys, but are also more vulnerable to poor behavioural outcomes in adolescence, which are likely to cascade into future difficulties (Haller et al. 2010).

The decreasing group, for boys, and the moderate group, for girls, were more socially disadvantaged in terms of parental income and living in social housing, and were more likely to have mothers who reported post-natal depressive symptoms than the low group. For girls, the moderate group was also more likely to have a single parent and mother who smoked during pregnancy, while the decreasing group, for boys, was more likely to live in a household with low educational qualifications. These two groups also included a higher proportion of BME children compared to the low or increasing trajectories. Few studies have examined the role of ethnicity in predicting internalizing trajectories from early childhood to adolescence, especially with an ethnically diverse population sample, so we have little information on how this finding might compare to previous studies. Given their relatively low levels of internalizing problems in adolescence, both of these groups were similar to the low group in terms of the adolescent-reported outcomes. Parents of girls, however, reported that their daughters had a higher probability of peer problems than the low group, which may highlight difficulties with social relationships.

In line with recent research (Booker et al. 2018), this study found that girls were more likely to use social media. However, social media use was not linked to trajectories of internalizing problems for any of the groups, for either gender. This finding may reflect recent research showing that moderate social media use does not predict changes in depressive symptoms, but rather increasing, excessive screen and media use relates to increasing depressive symptoms, highlighting that this relationship may be bidirectional (Houghton et al. 2018; Raudsepp and Kais 2019). Specific technology-based behaviours, such as social comparison and feedback seeking, have also been shown to be associated with depressive symptoms, suggesting a more nuanced approach to the study of adolescents’ media use (Nesi and Prinstein 2015). Early menarche was also not a risk factor for girls, supporting recent research showing that menarche status is not associated with worsening depression (McGuire et al. 2019). Rather, increases in depressive symptoms seem to be associated with physical changes that emerge early in the pubertal transition for early maturing girls, along with anticipatory concerns about social rejection.

## Limitations

There are a number of limitations to consider. First, internalizing problems were assessed on parental reports only, raising the problem of informant and methodological biases. It is also possible that parental reporting differed based on the gender of the child, contributing to potential biases. Second, the extent of our analyses is limited by the measures included in the multi-purpose longitudinal survey of a national cohort, many of which relied on a parsimonious measurement strategy. The use of SDQ, as a clinical screening tool, may also be a limitation. Although the SDQ (Goodman et al. 2000) is predictive of depression and other internalizing diagnoses, the trajectories themselves are not clinical. Furthermore, this broadly defined internalizing construct may be more stable over time than more distinct variations within this domain, such as separation anxiety and social anxiety, which may show different patterns of change over the course of development, with potential variation between genders (Carter et al. 2010; McLaughlin and King 2015). Third, as in all longitudinal studies, there was the problem of missingness in the data due to non-response for certain items or for a whole wave of data collection. This problem was addressed using MCS attrition weights and FIML estimation as implemented in STATA to adjust the likelihood function so that each case contributes information on the variables that are observed. Fourth, group-based trajectory analysis only provides a descriptive summary of a potential underlying typology in pathways. The fit indicators provide some guidelines about the number of types to select, and the final selection is based on consideration of parsimony, interpretability, BIC statistics, and average posterior probability of group membership. Individuals are discretely assigned to the best-fitting subgroup, despite some degree of imprecision in group membership. Lastly, only a subset of adolescent-reported outcomes was assessed and outcomes in late adolescence and adulthood are not yet available.

## Conclusions

This study offers insights into the development of internalizing problems for children and adolescents born in the new millennia. For boys and girls, there are two developmental trajectories demonstrating a high risk of clinically meaningful internalizing problems: a high pathway, exhibiting a high probability of internalizing problems from early childhood to adolescence and an increasing pathway, showing a heightened probability of internalizing problems before and during the pubertal transition. Given the recent attention placed on the internalizing problems of girls, one notable finding is the elevated percentage of boys on the high pathway, which is 1.75 times greater than the percentage of girls on a similar trajectory. Of further importance is the apparent increasing risk of internalizing problems for girls in adolescence, while this risk seems to level off for boys. Most concerning are the high levels of mental and physical health problems facing those on the high or increasing pathways. For example, in each of these two groups, more than one-third of the girls reported engaging in self-harm, which is twice the proportion of girls in the low or moderate groups, and approximately one-quarter of the boys and girls are overweight or obese in each of these two groups compared to about 15% in the lower problem groups. Girls on the increasing pathway reported an especially alarming level of problematic behaviours in adolescence including early sexual activity and more cannabis and cigarette use compared to the low group, confirming the “gender paradox of co-morbidity” (Loeber and Keenan 1994). Overall, these findings highlight that intervention strategies take a systemic view, targeting not only internalizing emotions, but also behaviours associated with health and well-being to circumvent the possibility of negative outcomes emerging in later adolescence and adulthood.

## References

[CR1] Achenbach, T. M. (1991). Integrative guide for the 1991 CBCL/4–18, YSR, and TRF profiles. Department of Psychiatry, University of Vermont.

[CR2] Angold A, Costello EJ, Messer SC, Pickles A, Winder F, Silver D (1995). The development of a short questionnaire for use in epidemiological studies of depression in children and adolescents. International Journal of Methods in Psychiatric Research.

[CR3] Barry CT, Sidoti CL, Briggs SM, Reiter SR, Lindsey RA (2017). Adolescent social media use and mental health from adolescent and parent perspectives. Journal of Adolescence.

[CR4] Bauer DJ, Curran PJ (2003). Distributional assumptions of growth mixture models: implications for overextraction of latent trajectory classes. Psychological methods.

[CR5] Best P, Manktelow R, Taylor B (2014). Online communication, social media and adolescent wellbeing: a systematic narrative review. Children and Youth Services Review.

[CR6] Booker CL, Kelly YJ, Sacker A (2018). Gender differences in the associations between age trends of social media interaction and well-being among 10-15 year olds in the UK. BMC Public Health.

[CR7] Burt, K. B., Douglas Coatsworth, J., & Masten, A. S. (2016). Competence and psychopathology in development. *Development and Psychopathology*, 1–50. 10.1002/9781119125556.devpsy409.

[CR8] Carter AS, Godoy L, Wagmiller RL, Veliz P, Marakovitz S, Briggs-Gowan MJ (2010). Internalizing trajectories in young boys and girls: the whole is not a simple sum of its parts. Journal of Abnormal Child Psychology.

[CR9] Chaiton MO, Cohen JE, O'Loughlin J, Rehm J (2009). A systematic review of longitudinal studies on the association between depression and smoking in adolescents. BMC Public Health.

[CR10] Cicchetti D, Rogosch FA (2002). A developmental psychopathology perspective on adolescence. Journal of Consulting and Clinical Psychology.

[CR11] Collishaw S, Maughan B, Natarajan L, Pickles A (2010). Trends in adolescent emotional problems in England: a comparison of two national cohorts twenty years apart. Journal of Child Psychology and Psychiatry.

[CR12] Costello DM, Swendsen J, Rose JS, Dierker LC (2008). Risk and protective factors associated with trajectories of depressed mood from adolescence to early adulthood. Journal of Consulting and Clinical Psychology.

[CR13] Croft S, Stride C, Maughan B, Rowe R (2015). Validity of the strengths and difficulties questionnaire in preschool-aged children. Pediatrics.

[CR14] Danzo S, Connell AM, Stormshak EA (2017). Associations between alcohol-use and depression symptoms in adolescence: examining gender differences and pathways over time. Journal of Adolescence.

[CR15] Dekker MC, Ferdinand RF, Van Lang ND, Bongers IL, Van Der Ende J, Verhulst FC (2007). Developmental trajectories of depressive symptoms from early childhood to late adolescence. Journal of Child Psychology and Psychiatry.

[CR16] Dockray S, Susman EJ, Dorn LD (2009). Depression, cortisol reactivity, and obesity in childhood and adolescence. Journal of Adolescent Health.

[CR17] Fanti KA, Henrich CC (2010). Trajectories of pure and co-occurring internalizing and externalizing problems from age 2 to age 12: findings from the National Institute of Child Health and Human Development study of early child care. Developmental Psychology.

[CR18] Fink E, Patalay P, Sharpe H, Holley S, Deighton J, Wolpert M (2015). Mental health difficulties in early adolescence: a comparison of two cross-sectional studies in England from 2009 to 2014. Journal of Adolescent Health.

[CR19] Goodman R (1997). The strengths and difficulties questionnaire (SDQ). Journal of Child Psychology and Psychiatry.

[CR20] Goodman R (2001). Psychometric properties of the strengths and difficulties questionnaire. Journal of the American Academy of Child & Adolescent Psychiatry.

[CR21] Goodman R, Ford T, Simmons H, Gatward R, Meltzer H (2000). Using the strengths and difficulties questionnaire (SDQ) to screen for child psychiatric disorders in a community sample. British Journal of Psychiatry.

[CR22] Green, H., McGinnity, A., Meltzer, H., Ford, T., & Goodman, R. (2005). *Mental health of children and young people in Great Britain*, 2004.

[CR23] Gutman LM, Joshi H, Parsonage M, Schoon I (2015). Children of the new century: Mental health findings from the millennium cohort study.

[CR24] Gutman LM, Joshi H, Parsonage M, Schoon I (2018). Trends in parent-and teacher-rated mental health problems among 10-and 11-year-olds in Great Britain: 1999–2012. Child and Adolescent Mental Health.

[CR25] Gutman LM, Joshi H, Parsonage M, Schoon I (2018). Gender-specific trajectories of conduct problems from ages 3 to 11. Journal of Abnormal Child Psychology.

[CR26] Gutman LM, Joshi H, Schoon I (2019). Developmental trajectories of conduct problems and cumulative risk from early childhood to adolescence. Journal of Youth and Adolescence.

[CR27] Haller M, Handley E, Chassin L, Bountress K (2010). Developmental cascades: linking adolescent substance use, affiliation with substance use promoting peers, and academic achievement to adult substance use disorders. Development and Psychopathology.

[CR28] Hansen K (2014). Millennium cohort study: A guide to the data sets.

[CR29] Hyde LW, Burt SA, Shaw DS, Donnellan MB, Forbes EE (2015). Early starting, aggressive, and/or callous–unemotional? Examining the overlap and predictive utility of antisocial behavior subtypes. Journal of Abnormal Psychology.

[CR30] Houghton S, Lawrence D, Hunter SC, Rosenberg M, Zadow C, Wood L, Shilton T (2018). Reciprocal relationships between trajectories of depressive symptoms and screen media use during adolescence. Journal of Youth and Adolescence.

[CR31] Johnson, J., Atkinson, M., & Rosenberg, R. (2015). Millennium Cohort Study: Psychological Developmental and Health Inventories Centre for Longitudinal Studies. http://www.cls.ioe.ac.uk/librarymedia%5Cdocuments%5CGuide%20to%20Psychological%20Inventories%20in%20MCS%282%29.pdf

[CR32] Jones BL, Nagin DS (2013). A note on a Stata plugin for estimating group-based trajectory models. Sociological Methods & Research.

[CR33] Joshi H, Fitzsimons E (2016). The millennium cohort study: the making of a multi-purpose resource for social science and policy. Longitudinal and Life Course Studies.

[CR34] Kelly Y, Zilanawala A, Sacker A, Hiatt R, Viner R (2016). Report: early puberty in 11-year-old girls: millennium cohort study findings. Children & Young People Now.

[CR35] Korhonen M, Luoma I, Salmelin RK, Helminen M, Kaltiala-Heino R, Tamminen T (2014). The trajectories of child’s internalizing and externalizing problems, social competence and adolescent self-reported problems in a Finnish normal population sample. School Psychology International.

[CR36] Letcher P, Smart D, Sanson A, Toumbourou JW (2009). Psychosocial precursors and correlates of differing internalizing trajectories from 3 to 15 years 1. Social Development.

[CR37] Leve LD, Kim HK, Pears KC (2005). Childhood temperament and family environment as predictors of internalizing and externalizing trajectories from ages 5 to 17. Journal of Abnormal Child Psychology.

[CR38] Lewis AJ, Kremer P, Douglas K, Toumborou JW, Hameed MA, Patton GC, Williams J (2015). Gender differences in adolescent depression: differential female susceptibility to stressors affecting family functioning. Australian Journal of Psychology.

[CR39] Loeber R, Keenan K (1994). Interaction between conduct disorder and its comorbid conditions: effects of age and gender. Clinical Psychology Review.

[CR40] McCrae N, Gettings S, Purssell E (2017). Social media and depressive symptoms in childhood and adolescence: a systematic review. Adolescent Research Review.

[CR41] McGuire TC, McCormick K, Koch MK, Mendle J (2019). Pubertal maturation and longitudinal trajectories of depression during early adolescence. Frontiers in Psychology.

[CR42] McLaughlin KA, King K (2015). Developmental trajectories of anxiety and depression in early adolescence. Journal of Abnormal Child Psychology.

[CR43] McLeod GFH, Horwood LJ, Fergusson DM (2016). Adolescent depression, adult mental health and psychosocial outcomes at 30 and 35 years. Psychological Medicine.

[CR44] Measelle JR, Stice E, Hogansen JM (2006). Developmental trajectories of co-occurring depressive, eating, antisocial, and substance abuse problems in female adolescents. Journal of Abnormal Psychology.

[CR45] Meltzer H, Gatward R, Goodman R, Ford T (2000). The mental health of children and adolescents in Great Britain.

[CR46] Moffitt TE, Harrington H, Caspi A, Kim-Cohen J, Goldberg D, Gregory AM, Poulton R (2007). Depression and generalized anxiety disorder: cumulative and sequential comorbidity in a birth cohort followed prospectively to age 32 years. Archives of General Psychiatry.

[CR47] Nagin D (2005). Group-based modeling of development.

[CR48] Nagin DS, Odgers C (2010). Group-based trajectory modeling in clinical research. Annual Review Clinical Psychology.

[CR49] Negriff S, Susman EJ (2011). Pubertal timing, depression, and externalizing problems: a framework, review, and examination of gender differences. Journal of Research on Adolescence.

[CR50] Nesi J, Prinstein MJ (2015). Using social media for social comparison and feedback-seeking: gender and popularity moderate associations with depressive symptoms. Journal of Abnormal Child Psychology.

[CR51] Nivard MG, Lubke GH, Dolan CV, Evans DM, Pourcain BS, Munafò MR, Middeldorp CM (2017). Joint developmental trajectories of internalizing and externalizing disorders between childhood and adolescence. Development and Psychopathology.

[CR52] Nolen-Hoeksema S, Girgus JS (1994). The emergence of gender differences in depression during adolescence. Psychological Bulletin.

[CR53] Patton GC, Olsson C, Bond L, Toumbourou JW, Carlin JB, Hemphill SA, Catalano RF (2008). Predicting female depression across puberty: a two-nation longitudinal study. Journal of the American Academy of Child & Adolescent Psychiatry.

[CR54] Public Health England (2016). The mental health of children in England.

[CR55] Raudsepp, L., & Kais, K. (2019). Longitudinal associations between problematic social media use and depressive symptoms in adolescent girls. *Preventive Medicine Reports, 100925*. 10.1016/j.pmedr.2019.100925 PMC6603436 31304081

[CR56] Richardson LP, Garrison MM, Drangsholt M, Mancl L, LeResche L (2006). Associations between depressive symptoms and obesity during puberty. General Hospital Psychiatry.

[CR57] Rodgers B, Pickles A, Power C, Collishaw S, Maughan B (1999). Validity of the malaise inventory in general population samples. Social Psychiatry and Psychiatric Epidemiology.

[CR58] Rutter M, Tizard J, Whitmore K (1970). Education, health and behavior.

[CR59] Schafer JL, Graham JW (2002). Missing data: our view of the state of the art. Psychological Methods.

[CR60] Shore L, Toumbourou JW, Lewis AJ, Kremer P (2018). Longitudinal trajectories of child and adolescent depressive symptoms and their predictors–a systematic review and meta-analysis. Child and Adolescent Mental Health.

[CR61] Skogen JC, Knudsen AK, Hysing M, Wold B, Sivertsen B (2016). Trajectories of alcohol use and association with symptoms of depression from early to late adolescence: the Norwegian longitudinal health behavior study. Drug and Alcohol Review.

[CR62] Sterba SK, Prinstein MJ, Cox MJ (2007). Trajectories of internalizing problems across childhood: heterogeneity, external validity, and gender differences. Development and Psychopathology.

[CR63] Strasburger VC, Hogan MJ, Mulligan DA, Ameenuddin N, Christakis DA, Cross C (2013). Children, adolescents, and the media. Pediatrics.

[CR64] Taylor MF, Brice J, Buckand N, Prentice-Lane E (2010). British household panel survey user manual volume a: Introduction, technical report and appendices.

[CR65] Toth, S. L., Petrenko, C. L., Gravener-Davis, J. A., & Handley, E. D. (2016). Advances in prevention science: a developmental psychopathology perspective. *Development and Psychopathology*, 1–59.

[CR66] Twenge JM, Joiner TE, Rogers ML, Martin GN (2018). Increases in depressive symptoms, suicide-related outcomes, and suicide rates among US adolescents after 2010 and links to increased new media screen time. Clinical Psychological Science.

[CR67] Ullsperger JM, Nikolas MA (2017). A meta-analytic review of the association between pubertal timing and psychopathology in adolescence: are there sex differences in risk?. Psychological Bulletin.

[CR68] von Eye A, Bergman LR (2003). Research strategies in developmental psychopathology: dimensional identity and the person-oriented approach. Development and Psychopathology.

[CR69] Woods HC, Scott H (2016). # Sleepyteens: social media use in adolescence is associated with poor sleep quality, anxiety, depression and low self-esteem. Journal of Adolescence.

[CR70] Zahn-Waxler C, Klimes-Dougan B, Slattery MJ (2000). Internalizing problems of childhood and adolescence: prospects, pitfalls, and progress in understanding the development of anxiety and depression. Development and Psychopathology.

[CR71] Zahn-Waxler C, Shirtcliff EA, Marceau K (2008). Disorders of childhood and adolescence: gender and psychopathology. Annual Review of Clinical Psychology.

